# Niche construction in quantitative traits: heritability and response to selection

**DOI:** 10.1098/rspb.2022.0401

**Published:** 2022-06-08

**Authors:** Laurel Fogarty, Michael J. Wade

**Affiliations:** ^1^ Max Planck Institute for Evolutionary Anthropology, Leipzig, Germany; ^2^ Indiana University, Bloomington, IN, USA

**Keywords:** niche construction, heritability, indirect genetic effects, breeding values

## Abstract

A central tenet of niche construction (NC) theory is that organisms can alter their environments in heritable and evolutionarily important ways, often altering selection pressures. We suggest that the physical changes niche constructors make to their environments may also alter trait heritability and the response of phenotypes to selection. This effect might change evolution, over and above the effect of NC acting via selection alone. We develop models of trait evolution that allow us to partition the effects of NC on trait heritability from those on selection to better investigate their distinct effects. We show that the response of a phenotype to selection and so the pace of phenotypic change can be considerably altered in the presence of NC and that this effect is compounded when trans-generational interactions are included. We argue that novel mathematical approaches are needed to describe the simultaneous effects of NC on trait evolution via selection and heritability. Just as indirect genetic effects have been shown to significantly increase trait heritability, the effects of NC on heritability in our model suggest a need for further theoretical development of the concept of heritability.

## 1. Introduction

A central tenet of niche construction (NC) theory is that organisms, through their own actions, can alter their environments in heritable and evolutionarily important ways. These changes can modify the selective pressures to which organisms are subject, sometimes substantially [1]. However, the physical changes niche constructors make to the environment might also change other important aspects of an organism's evolution. For example, NC might change the heritability of a trait expressed in an environment—that is, the ratio of the variance in heritable contributions to a phenotype to the phenotypic variance. Such changes to heritability have the potential to change the rates and perhaps trajectories of a species' evolution over and above the effects of NC acting via selection alone.

NC theory has a tradition of population genetic modelling. Heritability and related concepts, though, are typically modelled in a quantitative genetic framework [2]. Thus, the aim of this paper is twofold: first to allow direct comparisons between existing models of interacting traits and heritable environments and situate NC among those models. And second, to develop a framework which allows us to investigate aspects of NC that we currently lack the theory to understand, namely the effect of NC on breeding values, trait heritability and phenotypic change.

We suggest that NC has the potential to change selection on a trait, the structure of the fitness landscape, and the heritability of niche-constructed traits but that the contribution of each of these distinct alterations to the evolution process, in combination, are poorly understood. Although our focus emphasizes the effect of NC on breeding values and trait heritability, we point to other recent work on its effect on fitness (e.g. [3–5]) and note that our results strongly suggest that the effects of NC on heritability and selection interact (see Discussion), and that this interaction should be explored in future studies.

The model we present is related to other treatments of heritable environments or ‘extended phenotypes’ [6,7] such as those associated with maternal effects (e.g. [8,9]) sib effects and indirect genetic effects (IGEs), which have typically used quantitative genetic frameworks (e.g. [10–12]). Our model, too, draws on previous models of quantitative genetic evolution, particularly the work of Lande [13]. Consequently, it suffers from many of the same issues (see e.g. [14]) some of which may be compounded in the case of NC (see below). For example, these models typically assume that trait variances and covariances remain constant. This assumption is valid under some restrictive assumptions, the relaxation of which has been investigated elsewhere in detail (e.g. [15]). In our model, we also assume constant variances and covariances because using this framework allows us to compare models of correlated evolution (e.g. [13]), evolution in the presence of phenotypic interactions (e.g. [10]) and NC models directly.

Models of NC, including ours, are necessarily complex. They minimally include (i) a trait that alters or constructs an important resource in the environment, (ii) the ecological dynamics of this environmental resource, (iii) interactions between many, possibly unrelated, individuals and (iv) a phenotypic or selective response to environmental alterations. In NC systems, interactions between phenotypes need not be direct (i.e. focal individual with social partner) but rather can be indirect through modifications of a shared environmental resource, which is also modified simultaneously by abiotic forces. Moreover, NC effects can be trans-generational, and interactants need not coexist. Such temporally distant interactions can, in real systems, span a potentially large number of generations [16]. We note that some, but not all, of these elements are included in other theoretical frameworks, but not together. For example, in models of genotype-by-environment interaction, changing the environment can change the expression of a phenotype and thereby alter its heritability. As shown theoretically in [17] and empirically in [18] with IGEs, changing the intragenerational social environment can have similar effects. By contrast, models of parental and grandparental effects on offspring phenotypes are explicitly trans-generational. This class of models tends to emphasize how evolutionary rates change as the relationships between descendants and ancestors are discounted by genetic relatedness (e.g. [19]). The changes in selection caused by NC can be independent of relatedness, with all individuals in a single generation affected by the niche-constructing activities of individuals of prior generations regardless of relatedness. NC models typically include the ecological dynamics of resource renewal and degradation alongside the effects of the evolving niche-constructed traits. Thus, the models we derive below represent extensions of existing IGE theory that include elements unique to NC.

One of the most commonly cited and most studied forms of NC involves the creation and inheritance of an environmental resource to which all or many individuals in a population contribute and to which all or many individuals are sensitive. In this category we include, for example, modification of soils, stabilization of bedrock, alteration of nutrient cycling and socially constructed phenotypes such as the platform nests of some birds or the dams of beavers (see [16,20] for further examples and elaborations). This kind of NC forms the basis of many theoretical treatments of niche inheritance [16,20–23]. We note that it seems likely that different types of NC (as laid out by Odling-Smee *et al*. [16, p. 47]) affect selection and, perhaps, heritability in different ways. No one model addresses them all.

Finally, we draw on work motivated by the empirical observation that IGEs in laying hens had a much larger effect on desirable economic phenotypes than direct genetic effects (e.g. [24]). This work has shown, using the concept of ‘total breeding value’, how heritability in the presence of IGEs can be larger than heritability calculated in the absence of such interactions [25,26]. We investigate how the concept of total breeding value applies to NC models, where effects can extend to multiple past generations and between many interacting individuals. This allows us to identify differences between the IGE and NC theoretical approaches. In the sections that follow, we develop and compare the results of a general two-trait IGE model with our two-trait NC model and do so using a common quantitative genetic framework.

## 2. The models

There are a number of approaches to modelling social effects [17] that have demonstrated an equivalence between two common approaches: one founded on trait-based studies where a phenotype expressed by an individual is affected by its social partners, and another which is an extension of variance component analyses for heritability. In what follows, we describe the trait-based model of Moore *et al*. [10] and proceed to extend this model to include the effect of a niche-constructed resource. However, we note that other formulations are possible.

Following standard quantitative genetic models, we begin by partitioning individual phenotypic trait values, *z*, into two components: a heritable genetic component, *a*, and a non-heritable component, usually referred to as the ‘environmental deviation’, *e* [2]. Moore *et al*. [10] and others (e.g. [27]) introduce IGEs by further partitioning the environmental component, *e*, into a random, non-heritable component, *e_g_*, and a component, *e_z_*, that is affected by the trait value of another individual:

2.1 $$z=a+e_{g}+e_{z}.$$

Here, *e_z_* is determined by the trait value of another individual interacting with the individual with trait value *z*. Following Moore *et al.* [10], we label this other individual using a prime and substitute the trait value, $z^{\prime }\;=\;a{\;}^{\prime }+e{\;}^{\prime }$, in the other individual for *e_z_*. We also introduce the coefficient of $z^{\prime }$, *Ψ*, which measures the magnitude of the influence of $z^{\prime }$ on *z*. After this substitution, we have

2.2 $$z=a+e_{g}+\Psi z^{\prime }.$$

Moore *et al*. consider a number of cases including the simplest case of an interaction between two phenotypes (*z*_1_, *z*_2_) in two individuals (the focal individual and a social partner denoted by a prime) where just one of the phenotypes (*z*_1_) is affected by the social interaction and the other trait (*z*_2_) remains unchanged.

Moore *et al*. reconstruct equations (2.1) and (2.2) for two traits, 1 and 2, in a focal individual, with trait 2 modifying trait 1, and obtain

2.3 $$z_{1}=a_{1}+e_{1}+\Psi a_{2}^{\prime }+\Psi e_{2}^{\prime }$$

and

2.4 $$z_{2}=a_{2}+e_{2}.$$

Note that trait 2 is not affected by the social interaction. *Ψ* is the strength of the effect of trait 2 on the value of trait 1.

As Moore *et al*. point out, when individuals express interacting phenotypes, the breeding value, *A*, of an individual involves *both* the direct and indirect additive genetic values of the focal individual. Following Bijma [25], we define the total breeding value of an individual as the ‘direct and indirect average effects of its own genes'. ‘Average effect’ is defined by Fisher [28, p. 31] as ‘the amount of difference produced, on the average, in the total [trait value] of the population for each … gene substitution’. In the case of IGEs, this ‘average effect’ is a combination of the direct effect of a gene on the average phenotype and the indirect effect that an individual has on the trait value of their social partner or partners. For more details on the concept of total breeding value (see [25, p. 1351]) for a carefully worked-through example.

The response of the mean phenotype to selection ($\Delta \bar{z}$) on a trait can be calculated by modelling selection acting on the covariance between the trait value and the breeding value, so $\Delta \bar{z}=\mathrm{cov}(A,\;z)\beta$ for a single trait, where β is the selection differential defined in more detail below [2,10]. Applying this approach to two correlated traits and, labelling the genetic covariances between traits as *G_ij_*, where the indices *i* and *j* represent the traits, Moore *et al*. [10] get

2.5 $$\Delta {\bar{z}}_{1}=(G_{11}+\Psi G_{12})\beta_{1}+(G_{12}+\Psi G_{22})\beta_{2}.$$

Working similarly with *z*_2_, they get

2.6 $$\Delta {\bar{z}}_{2}=G_{22}\beta_{2}+G_{12}\beta_{1}.$$

Here, $\beta_{i}$ values are defined as the selection differential, *s*, divided by the phenotypic variance-co-variance matrix, **P**. Note that this implies that the strength of selection is a function of the phenotypic variance, which in turn is a function of the phenotype. Throughout our analyses, to focus on response to selection and changes to heritability, we consider $\beta_{i}$ values which are constants, but we note that this implies different values of *s* for different phenotypic variances. Focusing on IGEs, Wolf *et al*. [29] partitioned selection into selection differentials due to natural and social selection. This approach could be useful in developing models of NC in this framework that combine the effects of NC on selection and phenotype. We discuss in further detail the phenotypic variance for interacting phenotypes in the heritability section below. However, here, phenotypic and genetic variances are assumed to be constant over time as is typical of quantitative genetic models of trait evolution. This assumption has been criticized (see e.g. [14]) but holds up in the case of short-term evolution where selection is weak and a large number of loci contribute to the phenotypic trait, a property called the *constancy of the G matrix* [30]. For more complete detail on the construction of the IGE model above see [10] section entitled ‘interactions with non-reciprocal effects'. For the purposes of careful comparison, we derive a more general IGE model based on the Moore *et al*. model above which allows for an arbitrary number of interactants (*N* − 1 interactants, excluding the focal individual) of arbitrary relatedness (*r*) in electronic supplementary material, appendix A.

## 3. Ecological inheritance: the effect of previous generations

To be consistent with the bulk of existing theoretical literature describing NC, it is helpful to separate a niche-constructing trait from its effects on the environment. For this reason, we assume that a single trait is responsible for creating a resource and, subsequently, that resource has an effect on the expression of a second, different, trait. Population genetic NC models do this by considering the evolution of a niche-constructing locus alongside a resource-sensitive ‘recipient’ locus. Therefore, in developing a quantitative genetic model of NC, we describe the interaction between two co-evolving traits *z*_1_ and *z*_2_ as in the IGE model above, and in a manner consistent with previous work on NC. These two traits interact with one another through the niche-constructed resource instead of directly as in IGE models (e.g. [10]). This means that the relationship between the phenotype and the social partners can take different functional forms. This is similar to the type I–III ‘functional responses' of organisms to ecological resources, where the rate of intake of a resource (and in this case the phenotypic response to that resource) increases in different ways with resource availability [31]. In this paper, we model a linear relationship (i.e. a type I resource) but other formulations are possible.

Following Bijma's [25,26] treatment of groups of interacting individuals, we assume that the social structure remains unchanged from generation to generation, such that the number of individuals, related or not, with whom a focal individual interacts remains constant over time. We assume that the full population consists of a number of non-overlapping groups (interacting groups) of *N* interacting individuals, including the focal individual, with N≥2 for the NC model.

Finally, we incorporate another aspect of NC: ecological inheritance. NC theory models resources or artefacts manufactured by organisms that persist longer than the lifetime of the constructor and modify the selection experienced by descendants. These resources are experienced by descendants as an ‘ecological inheritance’ [16]. For instance, in the often-cited example of NC in earthworms, it is now well-established that earthworm soil processing generates ecological legacies that shape soil quality, productivity and community structure that persist for multiple generations (e.g. [32]). Ecological inheritance is both common and well documented [16]. This niche-constructed ‘heritable environment’ differs from the *e_i_* of most quantitative genetic models, which is assumed to be uncorrelated with genotype and to have a mean of 0. It is also different from the ‘common environment’ of families discussed, for example, by Falconer & Mackay [2]. There ‘common environment’ includes aspects of the rearing environment that contribute to phenotypic covariance (i.e. resemblance) between family members. Unlike organism-constructed environments, the ‘common environment’ is assumed to be reformed every generation, not inherited, and it is assumed not to be affected by trait expression in previous generations.

The niche-constructed ‘heritable environment’ also differs from the ‘heritable social environment’ discussed by Moore *et al*. [10]. Specifically, in IGE models, only the current social environment, i.e. the trait values of coexisting social partners, affects the evolution of trait z_1_. Trans-generational IGEs occur when mothers or grandparents are social partners [33–35]. In these models, heritable social environments must coexist with the focal individuals to permit the social partner to manifest the social behaviour; parental and grandparental effects involve direct relatives; they must vary among genotypes within populations to cause selection (e.g. different genotypes experience different maternal or sibling environments see, for example, equations (6) and (7) in [17]), and, their inter-generational scope is limited to one or two generations. Although, in principle, any number of generations could be included in some IGE models, the constraint of ‘coexistence’ limits the number of possible ancestral social partners and genetic relatedness discounts distant ancestral partners more strongly than more recent ones.

The ‘heritable environment’ relevant to NC theory is affected by the expression of a trait in one generation that persists *over a number of later generations*. The ‘social partners’ (of past generations) need not coexist with the current population members to exert an effect on present trait value or selection in the present generation. Here, we will assume that ecological ancestors are conspecifics but this could be extended to explore the role of NC in coevolution in the same way that IGE theory has been expanded to heterospecifics [27].

**Figure 1.**
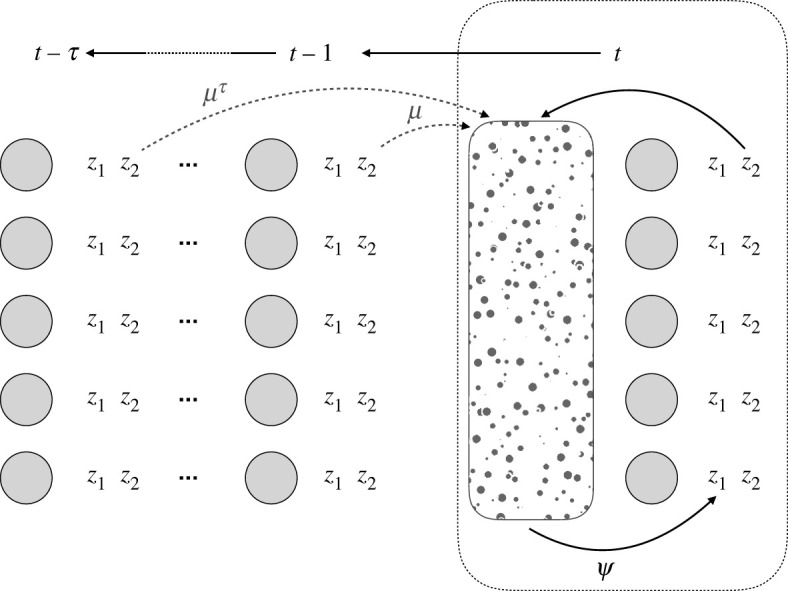
A schematic of the niche construction model including phenotypes *z*_1_ and *z*_2_, which have a persistent effect on a patchy resource landscape. This landscape in turn affects the expression of the phenotypes in the current generation. The patchy landscape implies that individuals can vary in the niche-constructed part of their phenotype, and that we do not model a mean group effect. The effect of the resource on the phenotype is modulated by the parameter *Ψ*. Time is denoted by value *t* and the time for which the effect of any single generation can persist is given by τ. We note that although we model a simple degradation of resource over time (modulated by μ), other formulations are possible (e.g. [16, pp. 387–389]). Similarly, more complex forms of *Ψ* would imply different functional relationships between resource and phenotype (e.g. [30]). (Online version in colour.)

We consider both genetic and ecological inheritance, so that an individual's offspring inherit its genes and its niche-constructed environment. This is modelled by allowing the expression of niche-constructing traits in previous generations to affect the value of a trait in a focal individual in the current generation, through the production of an otherwise decaying resource (figure 1). Thus, one possible form of the equation for the value of a quantitative resource-sensitive trait in a focal individual is

3.1 $$z_{1(t)}=a_{1(t)}+e_{1(t)}+\Psi \overset{N}{\underset{i=1}{\sum }}\overset{n}{\underset{\tau =0}{\sum }}\mu^{\tau }z_{2i(t-\tau )},$$

where μ reflects the time-dependent effect of an historically niche-constructed resource on the expression of the trait, *Ψ* describes the effect of the resource produced by trait 2 on trait 1 in.general, *t* is the current generation and τ indexes the previous *n* generations such that values with a subscript τ=2 reference values from two generations ago. In this case, μ discounts the effect of the expression of the trait in previous generations (meaning that abiotic forces erode the niche-constructed resource over time). Note that the form $\mu^{\tau }$ here means that we have assumed a ‘recency effect’ of the resource on the population. Different formulations might include a ‘primacy effect’ or an ‘equal weighting’ of each previous generation's NC on the current generation [16, pp. 387–388] or some more complex spatial or temporal form of resource degradation balanced with independent resource renewal (see [36]). The specific form of the discounting (represented here by $\mu^{\tau }$) is determined by the ecology of specific traits or systems. Whatever the process of discounting, change in resource level is inherently an ecological process, whose rate of change could well depend upon abiotic as well as biotic factors in the ecological community. Note also that we index the genetic and environmental contributions to the phenotype by generation. Again, this differs from standard quantitative genetic models, where the mean environmental contribution to a phenotype is assumed to be 0 at each generation and uncorrelated with genotypic contribution to a phenotype at each generation. Below, following the assumption of constancy of the G matrix discussed above, the variances contained in the G matrix are not indexed by time and are constant. It is clear from the formulation of the last term in equation (3.1) that ‘heritability of the environment’ in NC models is non-Mendelian, further distinguishing it from the heritable social environments discussed in IGE models.

For the niche-constructing trait (trait 2) that is not affected by the products of NC, we get

3.2 $$z_{2(t)}=a_{2(t)}+e_{2(t)}.$$

As before, we are interested in describing the per-generation evolutionary change in each trait. This involves calculating the heritable component of each trait or the total breeding values [25,26]. In this case, the breeding value is the same as it is in the IGE model above but includes the effect of the individual on *N* interactants, which can include the focal individual $\left(A=a_{1(t)}+N\Psi a_{2(t)}\right)$. Differently put, we assume that each individual has an effect on *N* individuals in a population by producing an amount of resource with effect *Ψ* which is used or experienced by those individuals.

We follow the method of [10] in describing the direct and correlated responses to selection in both traits as above. Therefore, for the change in *z*_1_ we obtain

3.3 $$\begin{array}{r} \Delta {\bar{z}}_{1}=\mathrm{cov}(a_{1(t)}+N\Psi a_{2(t)},z_{1(t)})\beta_{1}+\mathrm{cov}(a_{1(t)}+N\Psi a_{2(t)},z_{2(t)})\beta_{2}. \end{array}$$

The covariances in equation (3.3) require some unpacking. Expanding, we get

$$\begin{array}{rl} \Delta {\bar{z}}_{1} & =\mathrm{cov}\left(a_{1(t)}+N\Psi a_{2(t)},a_{1(t)}+e_{1(t)}+\Psi \overset{n}{\underset{\tau =0}{\sum }}\mu^{\tau }\overset{N}{\underset{i=1}{\sum }}z_{2,i,(t-\tau )}\right)\beta_{1}\\ & \;+\mathrm{cov}(a_{1(t)}+N\Psi a_{2(t)},a_{2(t)}+e_{2(t)})\beta_{2}. \end{array}$$

Taking just the covariances associated with the first term and using the assumption that environmental deviations are random, gives us

3.5 $$\begin{array}{rl} & \mathrm{cov}(a_{1(t)},a_{1(t)})+\mathrm{cov}\left(a_{1(t)},\Psi \overset{n}{\underset{\tau =0}{\sum }}\mu^{\tau }\overset{N}{\underset{i=1}{\sum }}a_{2,i,(t-\tau )}\right)\\ & \;+\mathrm{cov}\left(N\Psi a_{2(t)},\Psi \overset{n}{\underset{\tau =0}{\sum }}\mu^{\tau }\overset{N}{\underset{i=1}{\sum }}a_{2,i,(t-\tau )}\right). \end{array}$$

As above, we label $\mathrm{cov}(a_{1(t)},a_{1(t)})=G_{11}$ and $\mathrm{cov}(a_{1(t)},a_{2(t)})=G_{12}$. Where Moore *et al*. [10] assume that pairwise social interactions occur between unrelated individuals, we have assumed that the interacting group can include direct relatives and non-relatives so we must make assumptions about the extent of the relatedness between members of the interacting group as well as the change in relatedness over time. Relatedness is defined as $r\;=\;\mathrm{cov}(a,a^{\prime })/\mathrm{var}(a)$ [37]. In other words, relatedness between two interacting individuals is the correlation between their direct breeding values. Here we label the average relatedness between two randomly chosen members of the interacting group to be *r* (or equivalently assume that all members of the interacting group are equally related [38]. This can often be achieved experimentally, (e.g. [18]). We assume that *r* does not change over a short time (equivalent to the timescale on which the G matrix remains constant) and is created and maintained by some population genetic structure or other non-random assortment of breeding values [39]. However, the stability of *r* is a simplifying assumption and the more likely case of change in local relatedness over time could be modelled explicitly as it is, for example, in [40].

Because the current generation, *t*, includes the focal individual, we must consider the case where τ=0 (i.e. this generation) separately from cases where τ>0 (previous generations). Note that this means we are considering the focal individual separately from the *N* − 1 other interactants in the current generation. Taking the second term from equation (3.5) and considering the case where τ=0, such that $\mu^{\tau }=1$, we find that

3.6 $$\mathrm{cov}\left(a_{1(t)},\Psi \overset{N}{\underset{i=1}{\sum }}a_{2,i,(t)}\right)=\Psi G_{12}+(N-1)\Psi rG_{12},$$

and for τ>0 we get

$$\mathrm{cov}\left(a_{1(t)},\Psi \overset{n}{\underset{\tau =1}{\sum }}\mu^{\tau }\overset{N}{\underset{i=1}{\sum }}a_{2,i,(t)}\right)=N\Psi rG_{12}\overset{n}{\underset{\tau =1}{\sum }}\mu^{\tau }.$$

Treating the other terms in equation (3.5) similarly, we can now write an expression for $\Delta {\bar{z}}_{1}$ in terms of covariances:

3.7 $$\begin{array}{rl} \Delta {\bar{z}}_{1} & =(G_{11}\\ & \;+\Psi (NG_{12}+G_{12}+(N-1)rG_{12}+M_{1}NG_{12}r)\\ & \;+\Psi^{2}(NG_{22}+(N-1)NrG_{22}+M_{1}N^{2}rG_{22})\beta_{1}\\ & \;+(G_{12}+\Psi G_{22})\beta_{2}. \end{array}$$

where $M_{1}=\sum_{t=1}^{n}\mu^{\tau }$. Note that the response to selection now depends, not just on the interaction effect *Ψ*, but also on the number of interacting individuals, *N*, and the number of generations over which the effect of the niche-constructed resource persists, *n*, as well as on the average relatedness in the interacting group, *r*. Importantly, this NC model does not reduce to the IGE model above when *N* = 2 (i.e. when interactions are pairwise) because one's own niche-constructed products have an effect on the trait value, too.

## 4. Results

**Figure 2.**
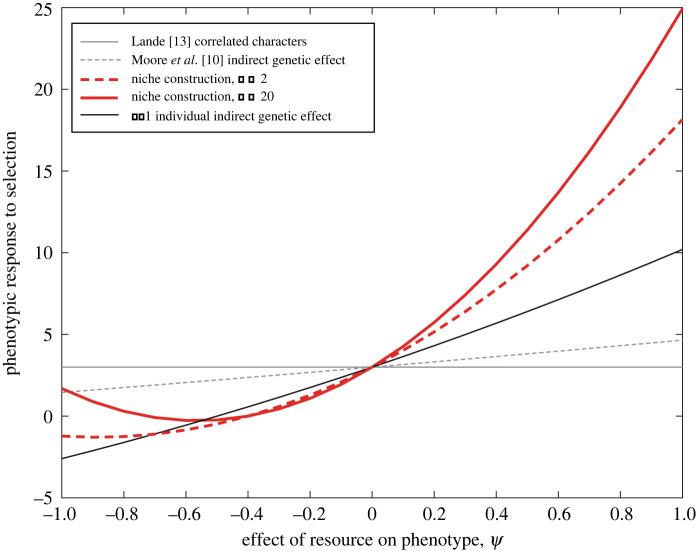
Response of phenotype to selection ($\Delta {\bar{z}}_{1}$) in the presence (red) and absence (black and grey) of niche construction. The solid grey line shows the correlated characters model [13]. This model shows the effect of correlated evolution in the absence of phenotypic interaction between traits. The dashed grey line shows the Moore *et al*.'s [10] model of a pairwise IGE. The black line shows the effect of group interactions in IGEs—here the focal individual interacts with *N* − 1 other individuals. This model is described in detail in the electronic supplementary material, appendix A. Finally, the niche construction model (thick red lines) for *n* = 2 (thick dashed red line) and *n* = 20 (thick solid red line) is plotted. This model includes a group interaction, the focal interacts with itself and *N* − 1 others, and ecological inheritance. Parameters are $\mu =0.85,\;G_{11}=4,\;G_{12}=2,\;G_{22}=1,\;\beta_{1}=\beta_{2}=0.5$, *N* = 5 and *r* = 0.1. Note that all models give the same result when Ψ=0 (i.e. when the interaction or resource has no effect on phenotype). (Online version in colour.)

In figure 2, we compare the response to selection in our NC model with that of the IGE model from Moore *et al.* [10], as well as with the simpler quantitative genetic model of correlated evolution without interactions from [13]. Note first that, when Ψ=0, all models give the same value for the response to selection, which is the value expected for correlated characters in the absence on an interaction. Figure 2 shows the effect of *Ψ* on the response to selection for the same parameters across all three models (note that the model of correlated characters from [13] does not include a phenotypic interaction between traits and so the response to selection is invariant with respect to *Ψ*). In the other models, in general, when *Ψ* is negative, organisms produce a resource or interaction effect that decreases the value of trait 1, leading to a reduced response to selection. Conversely, when *Ψ* is positive, an increased availability of the resource increases the value of trait 1, leading to an acceleration in the response to selection. The longer lasting the effects of NC (i.e. the greater the amount of ecological inheritance), the stronger the phenotypic response to selection.

Similar to the IGE model, the rate of phenotypic change can be considerably accelerated or decelerated by the effect of trait 2 on trait 1 in the NC model. However, the interaction effects in the NC model have a more profound effect on the response to selection than they do in the IGE model because in the former, they involve reciprocal interactions between a larger number of individuals who may interact without meeting directly and because the effects can be cumulative over generations. This causes the relationship between $\Delta {\bar{z}}_{1}$ and *Ψ* to be quadratic in the NC models, instead of linear as in the IGE model. Reciprocal interaction IGE models (fig. 3 in [10]) also generates a quadratic *Ψ*^2^ term, when the effect of *z*_1_ on *z*_2_ is equal in strength to the effect of *z*_2_ on *z*_1_. Note also that response to selection in the NC model relative to the IGE model can be substantially different depending on the form of μ and *r* (see electronic supplementary material, appendix D) and the values of *n*, *N*.

## 5. Heritability with indirect genetic effects and niche construction

Unlike IGE theory, NC theory includes explicit ecological models by accounting for multiple generations (*t* ≥ 2) and by accounting for non-genetic but heritable features of past environments (e.g. beaver dams and nesting sites). These environments do not themselves contain genes like the environments of social partners in IGE models. Rather, they are ecological products of past generations that remain associated with the descendants of a population (i.e. not just close relatives). Defining and understanding heritability in this case is a very hard problem because many things about traditional heritability break down in systems like this and, often, we cannot use the classic definition of heritability.

Typically, heritability is defined as the proportion of the total phenotypic variance that is attributable to variance in additive genetic effects [2]. Mathematically, narrow-sense heritability is classically written as

5.1 $$h^{2}=\frac{V_{A}}{V_{P}},$$

where *V_A_* is additive genetic variance (i.e. the covariance of the breeding value with itself) and *V_P_* is phenotypic variance (or, in other words, the covariance of the phenotypic value with itself). In the case of IGEs and NC, however, in addition to the additive genetic variance, the breeding value also contains a term that includes the individual's contribution to the IGE or niche-constructed environment. This has a number of important implications that change the nature of heritability and the kinds of questions that we can ask and answer about relationship between genetic variation and phenotypic variation.

Definitions of heritability that are equivalent in the absence of interactions change and are no longer equivalent in the presence of interactions. For example, absent interactions, the variance in breeding values is the same as the additive genetic variance, permitting heritability to be defined as either (i) the fractional contribution of additive genetic variance to the total variance, or, equivalently, (ii) the ratio of the variance in breeding values to the total variance, or, again equivalently, (iii) the ratio of the additive genetic component of phenotypic variance to the full phenotypic variance. With interactions defined by either IGEs or NC, the variance in the breeding values is not equal to the additive genetic variance. As a result, the definitions of heritability are no longer equivalent, nor are the genetic components of phenotypic variance equivalent to either of these previous values. To demonstrate this point, we contrast two definitions in the case of a simple IGE and then in the case of NC.

Consider the simple IGE described by equation (2.3) for interactions between unrelated individuals. First, we calculate the three relevant quantities: the additive genetic variance (*G*_11_), the phenotypic variance (*V_P_*) and the variance in breeding values (*V_A_*).

5.2 $$V_{A}=\mathrm{cov}(A,\;A)=\mathrm{cov}(a_{1}+\Psi a_{2},a_{1}+\Psi a_{2})$$

Taking the covariances, we get that

5.3 $$V_{A}=G_{11}+2\Psi G_{12}+\Psi^{2}G_{22}.$$

Similarly,

5.4 $$V_{p}=\mathrm{cov}(z,z)=G_{11}+E_{11}+\Psi^{2}G_{22}+\Psi^{2}E_{22}.$$

The three definitions of heritability are

5.5 $$h_{1}^{2}=\frac{G_{11}}{G_{11}+E_{11}+\Psi^{2}G_{22}+\Psi^{2}E_{22}},$$

5.6 $$h_{2}^{2}=\frac{G_{11}+2\Psi G_{12}+\Psi^{2}G_{22}}{G_{11}+E_{11}+\Psi^{2}G_{22}+\Psi^{2}E_{22}},$$

5.7 $$\;\mathrm{and}\;h_{3}^{2}=\frac{G_{11}+\Psi^{2}G_{22}}{G_{11}+E_{11}+\Psi^{2}G_{22}+\Psi^{2}E_{22}},$$

for the IGE with unrelated interactants (see electronic supplementary material, appendix C for $h_{3}^{2}$ with non-zero relatedness for an IGE). It is clear that the value of $h_{2}^{2}$ can be greater than 1 when the environmental variance is low and the genetic covariance between traits 1 and 2 is high, and where Ψ>0. Specifically, this happens when $2\Psi G_{12}>E_{11}+\Psi^{2}E_{22}$. A value of heritability greater than one is not compatible with the classic concept of heritability as the fraction of total variance attributable to genetic variance or indeed as the regression of breeding value on phenotypic value. This phenomenon has also been demonstrated for IGE models by Bijma & Wade [41], who pointed out that, when using the breeder's equation to describe the response to selection in a trait subject to IGEs, a value of *h*^2^ > 1 means that the response to selection can be extremely rapid. Their definition of heritability includes terms that represent the heritable variation present in the social environment as well as genetic variation within an individual [38].

For NC, we can calculate the values of the heritability measures $h_{1}^{2}$, $h_{2}^{2}$ and $h_{3}^{2}$ similarly (electronic supplementary material, appendices A and B) by first calculating the phenotypic variance in the NC model ($P_{nc}$; equation B.8, electronic supplementary material, appendix B), additive genetic variance (*G*_11_) and the variance in the breeding values ($G_{11}+2N\Psi G_{12}+\Psi^{2}N^{2}G_{22}$). This gives:

5.8 $$h_{1,nc}^{2}=\frac{G_{11}}{P_{nc}}\;,$$

5.9 $$h_{2,nc}^{2}=\frac{G_{11}+2N\Psi G_{12}+\Psi^{2}N^{2}G_{22}}{P_{nc}}$$

5.10 $$\;\mathrm{and}\;h_{3,nc}^{2}=\frac{G_{11}+2\Psi (G_{12}+(N-1)rG_{12}+M_{1}rNG_{12})+\Psi^{2}M_{1}^{2}(NG_{22}+N(N-1)rG_{22})}{P_{nc}}.$$

**Figure 3.**
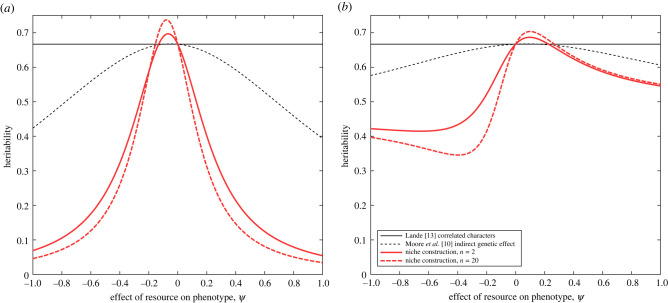
Comparison of two definitions of heritability given by (*a*) equation (5.5) and (*b*) equation (5.7). These are coincident in the absence of interactions (Ψ=0). Lines show heritability for the correlated characters model ([13], solid grey line), the IGEs model [10] and the niche construction model (thick red lines) for *n* = 2 (thick solid red line) and *n* = 20 (thick dashed red line) Parameters are $\mu =0.85,\;G_{11}=2,\;G_{12}=0.9,\;G_{22}=0.9,\;E_{11}=1,\;E_{22}=1,\;N=5,\;n=\{2,\;20\}$. (Online version in colour.)

Figure 3, we compare the IGE values with the NC values for two measures of heritability. Figure 3*a* shows $h_{1}^{2}$ and figure 3*b* shows $h_{3}^{2}$ for both models, allowing for non-zero relatedness. The challenge is how to properly define heritability for a trait where much of the potential heritable trait variation is not embodied in phenotypic variance of the individuals being measured and is not seen by selection every generation in the same way as additive genetic variation, for example, might be.

There is another important definitional difference to note between classical theory without interactions and that with IGEs or NC. One definition of breeding value given by Falconer & Mackay [2] is this: the deviation of the phenotypic mean of the progeny from the current phenotypic mean is the breeding value of the parents. This is not true in the case of multi-generational NC legacies (ecological inheritance) because the effect of the social environment on the mean phenotype of the progeny changes over time (because of the μ parameter). The deviation of the offspring from the current mean involves the breeding value of the parents *and* the contribution of the changing inherited environment. Ultimately, this implies that to fully understand the heritability and evolution of niche-constructed traits or indeed the amenability of such traits to change by artificial selection, we must understand the role of genetics alongside the complex and dynamic changes in the inherited environment caused by the niche-constructing activities of individuals. This involves both direct and indirect selection on the niche-constructing and niche-constructed traits and the way in which important aspects of the environment are constructed, accumulate and decay over time.

## 6. Discussion

The significance of the models presented above is twofold. (i) The models show that the pace of phenotypic change can be significantly different in the presence of niche-constructing interactions between genotypes and that this effect is compounded where interactions with previous generations are considered. This change in evolutionary dynamics is present independent of the effect of NC on fitness landscapes. (ii) The models show that the concept of heritability for traits defined by niche-constructing interactions and by IGEs contains terms that are not part of classic quantitative genetic theory.

The first point goes some way toward addressing the criticisms of NC as neither a novel nor a useful concept in evolutionary biology. Critics have argued that proponents of NC theory conflate processes that generate variation on which selection can act with processes that do not (e.g. [42–44]). However, our model illustrates that NC's influence on evolution works in three ways. First, NC can plastically change the value of an expressed trait, changing the fitness of an individual without changing the fitness landscape or the breeding values associated with that trait (e.g. increased body size resulting from constructing a higher quality environment). Second, NC can alter the fitness landscape, in other words, it can change the selection pressures to which an individual is subject without changing the breeding values associated with the trait (e.g. large body size may be favoured in resource-rich constructed environments but small body size in the absence of NC). This is the way NC is most often characterized but, as our model hopefully shows, this may rarely be its only effect. And finally, NC can change breeding values, thereby changing the nature of trait heritability (e.g. the heritability of body size depends not only on transmission of genes across generations but also on the ecological legacy of modified environmental conditions).

We argue that these distinctions are important. Dawkins [42], following Sterelney [45], suggested that there should be a division between what they dubbed ‘niche changing’ and ‘NC’ proper. In this line of argument, niche changing includes changes to the environment that are passive by-products of an organism's way of life while ‘NC’ includes any environmental changes that are ‘actively engineered’. Dawkins asserted that ‘niche changing’ does not generate covariances between niche-constructing and niche-constructed genotypes and phenotypes and thus cannot be considered evolutionarily important to the same degree (see also [43,44]).

Using the models above and incorporating insights from other models of NC, we can directly address this point. The breeder's equation describes the evolutionary change in a trait over a single generation. As pointed out by Brodie [43], the engine that drives this evolutionary change is covariance between phenotypes and genotypes, and phenotypes and fitness. More concretely, as per Brodie [43], the evolutionary response can be written as

5.11 $$\Delta {\bar{z}}_{1}=h^{2}s=\mathrm{cov}(A,\;A)\mathrm{cov}\left(\frac{W(z)}{\bar{W}},\;z\right)P^{-1}.$$

Choosing one definition of *h*^2^ (see above) and expanding the breeder's equation we can see that the evolutionary response of a trait can be altered through changes in phenotypes (*z*-values), fitness landscapes (*W*(*z*)) and/or breeding values (*A*). Note that our models here have dealt, mainly, with changes to phenotypes, breeding values and phenotypic variance. Other models (e.g. [5]) have explored the relationship between NC and fitness landscapes. Here, we suggest that these different effects of NC separately or in combination produce a continuum from ‘weak’ to ‘strong’ NC, rather than a qualitative difference between ‘niche changing’ and ‘NC’. By this, we mean that types of NC range from those that only change phenotype, to those that change phenotypes, fitness landscapes and breeding values, simultaneously. All points on the spectrum from weak to strong NC have systematic and potentially important effects on trait evolution.

Of course, it remains to be understood how often the processes described here are important in real populations. However, most critics of NC theory have not disputed that it occurs, nor that it generates complex interactions between traits, both within and between species. Instead, they have challenged the interpretation of such interactions. We believe that the results of our NC model and current evidence favour arguments supporting an important role for NC in evolution. We have shown that, in theory, such interactions can have a strong impact on heritability, not just selection. Evidence from IGEs and their effects on heritability is growing, while the possibly great effects owing to NC have rarely been measured. In regard to IGEs and heritability, research on domestic chickens suggests that IGEs could account for up to 87% of heritable variation in survival found in crossbred chickens [24]. For NC, recent experimental studies of maternal and larval NC in dung beetles, show robust influences on offspring traits, fitness and heritability [46–48].

There may be some potentially serious consequences of failing to extend the concept of heritability to include the effects of ecological inheritance and NC. Where calculations of the response to selection are economically or ecologically important such as in agricultural breeding programmes or conservation contexts, it is important to note that the accuracy of estimates will depend on the strength of NC or IGEs in the system. For example, it may not be possible to estimate heritability from parent–offspring regressions nor are such regressions useful guides to the response to selection as they are in the absence of interactions. This does not suggest that parent–offspring and other family resemblances cannot be calculated; we argue, instead that in theory they do not predict the response to selection as they do in systems without such interactions.

One application of more comprehensive models may be in efforts to conserve endangered species. In this context, maintaining genetic diversity has been identified as a key aim which would help to maintain the resilience of species to rapid environmental change. As we have seen from the models above, in niche-constructing species, much of the potential diversity in a trait is not contained in the genomes of individuals but in the altered, inherited environment. This may mean that efforts to preserve genetic diversity isolated from efforts to conserve the environment in which a species has evolved and engineered its niche, risks missing huge wells of potential adaptability and response to change. This has consequences for our understanding of, for example, zoo breeding programmes as well as reintroductions and other conservation efforts that do not include the inherited environment. For example, it is already well-known (e.g. [49]) that reconstruction of the pre-agricultural microbial community is essential for tallgrass prairie restoration. This underlines the importance of conserving species *in habitats* as the potential reservoirs of resilience to change for many species and NC theory illustrates why such co-evolved habitats are crucial. The measurement of the contribution of IGEs to variance within populations may provide a framework with which to test the same in the context of NC. In the IGE sphere, cross-fostering is essential to measuring IGEs. Other methods like transplanting a population to a new environment (without cross-fostering) might allow researchers to measure the size of the effect of niche-constructed ancestry. However, transplanting would not give a complete picture of how that effect comes to be inherited.

The analyses presented in this paper draw out differences, some substantial, between models reliant solely on processes deemed core to the ‘modern synthesis’ and those that additionally incorporate a process emphasized by the extended evolutionary synthesis, namely the evolutionary feedbacks central to NC. Minimally, they suffice to establish that the phenomena represented by the concept of NC are not already fully understood through conventional quantitative genetics models. Rather, it is clear that there are gaps in our understanding of how evolution proceeds in systems where complex indirect interactions, or multi-generational effects mediated by ecological legacies and evolutionary feedbacks, are dominant. How important these differences are expected to be in any given system is, of course, an empirical question—but it is one that will not, and cannot, be answered in the absence of a complete evolutionary theory.

## Data Availability

Additional data are provided in the electronic supplementary material [50].

## References

[1] Clark AD, Deffner D, Laland K, Odling-Smee J, Endler J. 2019 Niche construction affects the variability and strength of natural selection. Am. Nat. **195**, 16-30. 31868536 10.1086/706196

[2] Falconer DS, Mackay T. 1996 Introduction to quantitative genetics. London, UK: Longman Group.

[3] Laland K, Matthews B, Feldman MW. 2016 An introduction to niche construction theory. Evol. Ecol. **30**, 191-202. (10.1007/s10682-016-9821-z) 27429507 PMC4922671

[4] Kylafis G, Loreau M. 2008 Ecological and evolutionary consequences of niche construction for its agent. Ecol. Lett. **11**, 1072-1081. (10.1111/j.1461-0248.2008.01220.x) 18727670

[5] Tanaka MM, Godfrey-Smith P, Kerr B. 2020 The dual landscape model of adaptation and niche construction. Phil. Sci. **87**, 478-498. (10.1086/708692)

[6] Dawkins R. 1982 The extended phenotype. Oxford, UK: WH Freeman.

[7] Bailey NW. 2012 Evolutionary models of extended phenotypes. Trends Ecol. Evol. **27**, 561-569. (10.1016/j.tree.2012.05.011) 22832012

[8] Eisen EJ. 1967 Mating designs for estimating direct and maternal genetic variances and direct-maternal genetic covariances. Can. J. Genet. Cytol. **9**, 13-22. (10.1139/g67-002) 5616732

[9] Riska B, Atchley WR, Rutledge JJ. 1984 A genetic analysis of targeted growth in mice. Genetics **107**, 79-101. (10.1093/genetics/107.1.79) 6724298 PMC1202316

[10] Moore AJ, Brodie ED, Wolf JB. 1997 Interacting phenotypes and the evolutionary process. I. Direct and indirect genetic effects of social interactions. Evolution **51**, 1352-1362. (10.1111/j.1558-5646.1997.tb01458.x) 28568644

[11] Wolf JB, Brodie ED, Cheverud JM, Moore AJ, Wade MJ. 1998 Evolutionary consequences of indirect genetic effects. Trends Ecol. Evol. **13**, 64-69. (10.1016/S0169-5347(97)01233-0) 21238202

[12] Wolf JB. 2000 Indirect genetic effects and gene interactions. In Epistasis and the evolutionary process (eds JB Wolf, ED Brodie III, MJ Wade), pp. 158-176. Oxford, UK: Oxford University Press.

[13] Lande R. 1979 Quantitative genetic analysis of multivariate evolution, applied to brain: body size allometry. Evolution **33**, 402-416. (10.1111/j.1558-5646.1979.tb04678.x) 28568194

[14] Turelli M. 1988 Phenotypic evolution, constant covariances, and the maintenance of additive variance. Evolution **42**, 1342-1347. (10.1111/j.1558-5646.1988.tb04193.x) 28581080

[15] Gilpin W, Feldman MW. 2019 Cryptic selection forces and dynamic heritability in generalized phenotypic evolution. Theor. Popul. Biol. **125**, 20-29. (10.1016/j.tpb.2018.11.002) 30528351

[16] Odling-Smee J, Laland KN, Feldman MW. 2003 Niche construction: the neglected process in evolution. Princeton, NJ: Princeton University Press.

[17] McGlothlin JW, Brodie ED. 2009 How to measure indirect genetic effects: the congruence of trait-based and variance-partitioning approaches. Evolution **63**, 1785-1795. (10.1111/j.1558-5646.2009.00676.x) 19245673

[18] Bleakley BH, Brodie III ED. 2009 Indirect genetic effects influence antipredator behavior in guppies: estimates of the coefficient of interaction *psi* and the inheritance of reciprocity. Evolution **63**, 1796-1806. (10.1111/j.1558-5646.2009.00672.x) 19245394

[19] Cheverud JM, Moore AJ. 1994 Quantitative genetics and the role of environment provided by relatives in behavioural evolution. In Quantitative genetic studies of behavioural evolution (ed. CRB Boake), pp. 67-100. Chicago, IL: University of Chicago Press.

[20] Gurney WSC, Lawton JH. 1996 The population dynamics of ecosystem engineers. Oikos **76**, 273. (10.2307/3546200)

[21] Laland KN, Odling-Smee FJ, Feldman MW. 1996 The evolutionary consequences of niche construction: a theoretical investigation using two-locus theory. J. Evol. Biol. **9**, 293-316. (10.1046/j.1420-9101.1996.9030293.x)

[22] Kylafis G, Loreau M. 2011 Niche construction in the light of niche theory. Ecol. Lett. **14**, 82-90. (10.1111/j.1461-0248.2010.01551.x) 21073644

[23] Rendell L, Fogarty L, Laland KN. 2011 Runaway cultural niche construction. Phil. Trans. R. Soc. B **366**, 823-835. (10.1098/rstb.2010.0256) 21320897 PMC3048990

[24] Peeters K, Eppink TT, Ellen ED, Visscher J, Bijma P. 2012 Indirect genetic effects for survival in domestic chickens (*Gallus gallus*) are Magnified in crossbred genotypes and show a parent-of-origin effect. Genetics **192**, 705-713. (10.1534/genetics.112.142554) 22851648 PMC3454891

[25] Bijma P. 2011 A general definition of the heritable variation that determines the potential of a population to respond to selection. Genetics **189**, 1347-1359. (10.1534/genetics.111.130617) 21926298 PMC3241417

[26] Bijma P. 2014 The quantitative genetics of indirect genetic effects: a selective review of modelling issues. Heredity **112**, 61-69. (10.1038/hdy.2013.15) 23512010 PMC3860160

[27] De Lisle SP, Bolnick DI, Brodie ED, Moore AJ, McGlothlin JW. 2022 Interacting phenotypes and the coevolutionary process: interspecific indirect genetic effects alter coevolutionary dynamics. Evolution **76**, 429-444. 34997942 10.1111/evo.14427 PMC9385155

[28] Fisher RA. 1999 The genetical theory of natural selection. Oxford, UK: Oxford University Press.

[29] Wolf JB, Brodie ED, Moore AJ. 1999 Interacting phenotypes and the evolutionary process. II. Selection resulting from social interactions. Am. Nat. **153**, 254-266. (10.1086/303168) 29585974

[30] Jones AG, Arnold SJ, Burger R. 2004 Evolution and stability of the G-matrix on a landscape with a moving optimum. Evolution **58**, 1639-1654. (10.1111/j.0014-3820.2004.tb00450.x) 15446419

[31] Holling CS. 1959 The components of predation as revealed by a study of small-mammal predation of the European Pine Sawfly. Can. Entomol. **91**, 293-320. (10.4039/Ent91293-5)

[32] Frelich LE et al. 2019 Side-swiped: ecological cascades emanating from earthworm invasions. Front. Ecol. Environ. **17**, 502-510. (10.1002/fee.2099) 31908623 PMC6944502

[33] Kirkpatrick M, Lande R. 1989 The evolution of maternal characters. Evolution **43**, 485-503. (10.1111/j.1558-5646.1989.tb04247.x) 28568400

[34] Prizak R, Ezard THG, Hoyle RB. 2014 Fitness consequences of maternal and grandmaternal effects. Ecol. Evol. **4**, 3139-3145. (10.1002/ece3.1150) 25247070 PMC4161186

[35] Dury GJ, Wade MJ. 2020 When mother knows best: a population genetic model of transgenerational versus intragenerational plasticity. J. Evol. Biol. **33**, 127-137. (10.1111/jeb.13545) 31549475 PMC7891633

[36] Lehmann L. 2008 The adaptive dynamics of niche constructing traits in spatially subdivided populations: evolving posthumous extended phenotypes. Evolution **62**, 549-566. (10.1111/j.1558-5646.2007.00291.x) 17983464

[37] Queller DC. 1992 A general model for kin selection. Evolution **46**, 376-380. (10.1111/j.1558-5646.1992.tb02045.x) 28564031

[38] Bijma P, Muir WM, Van Arendonk JAM. 2007 Multilevel selection 1: quantitative genetics of inheritance and response to selection’. Genetics **175**, 277-288. (10.1534/genetics.106.062711) 17110494 PMC1775021

[39] McGlothlin JW, Moore AJ, Wolf JB, Brodie ED. 2010 Interacting phenotypes and the evolutionary process. III. Social evolution. Evolution **64**, 2558-2574. (10.1111/j.1558-5646.2010.01012.x) 20394666

[40] Lehmann L. 2007 The evolution of trans-generational altruism: kin selection meets niche construction. J. Evol. Biol. **20**, 181-189. (10.1111/j.1420-9101.2006.01202.x) 17210011

[41] Bijma P, Wade MJ. 2008 The joint effects of kin, multilevel selection and indirect genetic effects on response to genetic selection. J. Evol. Biol. **21**, 1175-1188. (10.1111/j.1420-9101.2008.01550.x) 18547354

[42] Dawkins R. 2004 Extended phenotype—but not too extended (reply to Laland, Turner, Jablonka). Biol. Phil. **19**, 377-396. (10.1023/B:BIPH.0000036180.14904.96)

[43] Brodie III ED. 2005 Caution: niche construction ahead. Evolution **59**, 249-251. (10.1554/BR05-1)

[44] Scott-Phillips TC et al. 2013 The niche construction perspective: a critical appraisal. Evol. Int. J. Org. Evol. **68**, 1231-1243. 10.1111/evo.12332 PMC4261998 24325256

[45] Sterelny K. 2001 Niche construction, developmental systems, and the extended replicator. In Cycles of contingency: developmental systems and contingency (eds S Oyama, RD Gray, PE Griffiths), pp. 333-349. Cambridge, MA: MIT Press.

[46] Schwab DB, Riggs HE, Newton ILG, Moczek AP. 2016 Developmental and ecological benefits of the maternally transmitted microbiota in a dung beetle. Am. Nat. **188**, 679-682. (10.1086/688926) 27860508

[47] Schwab DB, Casasa S, Moczek AP. 2017 Evidence of developmental niche construction in dung beetles: effects on growth, scaling and reproductive success. Ecol. Lett. **20**, 1353-1363. (10.1111/ele.12830) 28942603

[48] Dury GJ, Moczek AP, Schwab DB. 2020 Maternal and larval niche construction interact to shape development, survival, and population divergence in the dung beetle *Onthophagus taurus*. Evol. Dev. **22**, 358-369. (10.1111/ede.12348) 33448595

[49] Fierer N, Ladau J, Clemente JC, Leff JW, Owens SM, Pollard KS, Knight R, Gilbert JA, McCulley RL. 2013 Reconstructing the microbial diversity and function of pre-agricultural tallgrass prairie soils in the United States. Science **342**, 621-624. (10.1126/science.1243768) 24179225

[50] Fogarty L, Wade MJ. 2022 Niche construction in quantitative traits: heritability and response to selection. *Figshare*. (10.6084/m9.figshare.c.5994510) PMC9156914 35642369

